# Lensing in the Ultrasonic Domain using Negative Refraction Induced by Material Contrast

**DOI:** 10.1038/s41598-019-42655-3

**Published:** 2019-04-24

**Authors:** C. T. Manjunath, Prabhu Rajagopal

**Affiliations:** Centre for Nondestructive Evaluation and Department of Mechanical Engineering, Indian Institute of Technology Madras, Chennai, Tamil Nadu 600036 India

**Keywords:** Mechanical engineering, Acoustics

## Abstract

The focusing of ultrasound using topographic lenses, typically made of plates with step changes that cause an interaction between forward- and backward-propagating guided waves, has been widely studied in recent years. However, such ‘step-change’ lenses require precise machining and moreover, the thick-thin structure can be unstable during deployment in practical inspection applications. The work reported here follows from the insight that perhaps any approach to induce a mismatch in acoustical impedance as achieved by the step-change can also lead to focusing of ultrasonic guided waves. By carefully choosing the impedance pairing, a novel material contrast lens stacking Aluminium and Molybdenum plates in series is shown to achieve focusing of ultrasound through negative refraction. The interface between the two metals causes the interaction of the forward-propagating second symmetric Lamb mode S_2_ into the backward- propagating first symmetric S_2b_. The focusing of Lamb waves is demonstrated using numerical simulations validated by experiments. Comparison with a simple Aluminium-Aluminium plate combination brings out the underlying physics of focusing using the proposed material contrast lens. Simulation results showing super-resolution imaging using the proposed material contrast lens  are also presented, demonstrating the power of the proposed approach. This report opens up the possibilities of developing new lensing devices for use in medical imaging and nondestructive evaluation, among other possible applications.

## Introduction

Techniques to improve the resolution of imaging systems beyond the diffraction limit^1–4^ been of much interest in the recent years. Phononic crystals^5–7^ and metamaterials^8–10^ are examples of such approaches to achieve subwavelength imaging and super-resolution in various domains. Focusing of acoustic and elastic waves have been demonstrated through lenses made of phononic crystals^11–16^ and holey metamaterials^17–22^. However, typically the process to design and manufacture such lenses is complex. Hence, researchers have proposed simpler and cost effective approaches that can produce lenses of similar quality. Ultrasonic guided wave based lenses making use of the phenomenon of negative refraction (NR) have been proposed^23,24^ to show how focusing can be achieved due to thickness changes in waveguides. Obtaining a narrow focal spot is important to overcome the diffraction limit in applications related to medical and industrial imaging. To date, multiple investigations have been reported in the domain of acoustics^25–27^ in order to characterize and reduce such focal spot aberrations.

Metamaterial concepts have also been proposed in the ultrasonic regime. However, due to the presence of coupled modes of wave propagation in elastic solids^28^, concepts developed for acoustics are not entirely portable to ultrasonics. This paper explores a different route to achieve super-resolution using waveguide-based topographic lenses for ultrasonics. The existence of backward wave propagation in elastic waveguides in the form of plates, has been studied with much interest in recent years^29,30^ and this forms the basis for the ‘topographic’ or ‘waveguide lenses’. Waveguide lenses are much simpler to design, easy to fabricate and are relatively inexpensive.

Lamb waves are guided elastic waves propagating in plate -type waveguides. They are in general dispersive and multimodal in nature and are governed by the  Rayleigh-Lamb equations^28,31^. Depending on the particle vibration with respect to the mid-plane of the plate thickness, Lamb waves are classified as symmetric and anti-symmetric modes based on the following relations,

1 $$\frac{\tan (qh)}{\tan (ph)}=-\,{[\frac{4{k}^{2}pq}{{({q}^{2}-{k}^{2})}^{2}}]}^{+1}\,{\rm{for}}\,{\rm{symmetric}}\,{\rm{modes}}$$

2 $$\frac{\tan (qh)}{\tan (ph)}=-\,{[\frac{4{k}^{2}pq}{{({q}^{2}-{k}^{2})}^{2}}]}^{-1}\,{\rm{for}}\,\mathrm{anti}-\mathrm{symmetric}\,{\rm{modes}}$$

where ${p}^{2}=\frac{{\omega }^{2}}{{c}_{L}^{2}}-{k}^{2}$ and ${q}^{2}=\frac{{\omega }^{2}}{{c}_{T}^{2}}-{k}^{2}$, and c_L_ and c_T_ are the longitudinal and transverse velocities respectively; k and *ω* is the wave number and circular frequency respectively; 2 h is the plate thickness.

Lamb waves exhibit an interesting phenomenon in the form of ‘backward wave propagation’, where some of the higher-order modes at certain frequencies have phase velocity (c_ph_) in the direction opposite to their group velocity (c_gr_). An experimental demonstration of the focusing of  Lamb waves using a plate waveguide with a thickness change was discussed in^23^. The study showed that the forward-propagating mode (S_2_) gets mode converted to backward- propagating (S_2b_) mode at the interface between the plates with two different thicknesses. On similar lines, ultrasound focusing using a double step change was also demonstrated^24^. A topographic lens made of thin metallic slices was proposed to achieve the focusing of the fundamental anti-symmetric (A_0_) mode^32^. Researchers^33–35^ have reported that such focusing can also be achieved by mode conversion occurring at plate edges using negative reflection of Lamb waves.

The key insight that led to the work reported here, is the fact that focusing in waveguides with step changes^23,24^, involves an acoustic impedance mismatch. The refraction of backward-propagating Lamb waves at an interface on a plate is generated at a given propagation frequency and wave number. This happens because the plate also generates a forward- propagating mode at the same frequency and wave number. We believe that a similar result can also be achieved by any mechanism whereby an acoustic impedance mismatch can be generated at an interface. In order to do this, here we propose to combine two different materials to form a lens (referred to hereafter in the present article as a ‘Material Contrast lens’). The advantage with this approach is that with the same thickness on both sides of the interface, precise machining to obtain step changes can be avoided. While this approach could have an issue in the form of focal-spot aberrations, these are perhaps artifacts of any method that induces abrupt changes in acoustic impedance across an interface, and similar effects have also been observed with Step Change lenses^23,24^. While a detailed consideration of and mitigation of such effects is beyond the scope of this report intended as a first presentation of this concept, some possible approaches to this are discussed later (see Discussion section).

In this report, we experimentally demonstrate the capability of the proposed material contrast lens, to focus ultrasound. Material selection and design of the lens are discussed in detail in the following section. Results from experimental scans are then presented clearly showing the focusing of ultrasound due to the proposed material contrast lens. The paper then discusses results obtained using experimentally validate numerical simulations, demonstrating super-resolution imaging using the proposed lens and concludes with directions for further work.

## Results

### Design of Material Contrast lens

**Figure 1:**
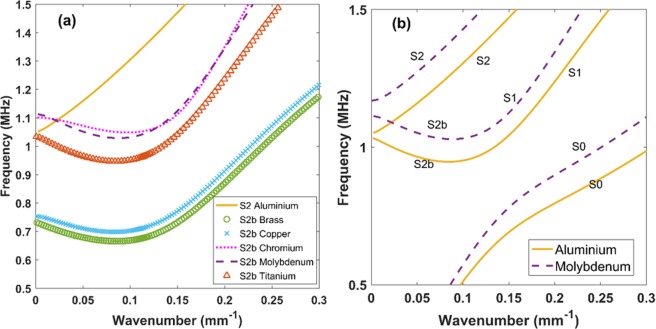
Lamb wave dispersion curves generated for a plate thickness of 3 mm. **(a)** Illustration of the S_2_ mode of Aluminium and S_2b_ mode of the other materials. **(b)** Intersection of S_2_ and S_2b_ mode of Aluminium and Molybdenum respectively at 1.09 MHz frequency.

One of the important factors influencing the design and performance of the proposed lens is the material selection. Here, the materials comprising the lens were chosen such that, the conversion of forward wave (S_2_) to backward wave (S_2b_) occurs at the same frequency-wave number combination, in both of them. In order to observe this, dispersion curves were generated using DISPERSE^36,37^ for some commonly used metallic materials (see Fig. 1). Aluminium was preferred as the first material (referred to hereafter as the ‘parent material’) owing to its easy availability and affordability. Five other commonly available metallic materials were chosen - Brass, Chromium, Copper, Molybdenum and Titanium. The second material was selected based on the extent of mode conversion undergone by the S_2_ mode in the parent metal into S_2b_ mode upon crossing the interface. From Fig. 1(a), we observe that the S_2_ mode in Aluminium intersects with the S_2b_ only in Molybdenum and Chromium. A close-up view of the intersection of the S_2_ mode in Aluminium and the S_2b_ mode in Molybdenum is shown in Fig. 1(b).

Based on the aforementioned match in the frequency-wavenumber factor, Molybdenum was selected as the second material for the lens bceause of its easier availability and machinability. Before the fabrication of the lens, the mechanical properties of the selected materials were determined through conventional  bulk ultrasonic pulse-echo testing. These values were found to closely match with the ones reported in literature (tabulated below)^38^. The material property values listed in Table 1. were used for all further calculations.

**Table 1:** Mechanical properties of materials selected for the fabrication of Material Contrast lens, as obtained using conventional bulk ultrasonic pulse-echo testing.

Material	Mechanical Properties		
	Elastic Modulus E (GPa)	Density ρ (kg/m^3^)	Poisson’s ratio µ
Aluminium	70	2700	0.34
Molybdenum	324	10240	0.29

### Numerical Simulation

At the selected frequency of 1.09 MHz, in-plane excitation was given on top of the plate, which generates all possible in-plane modes. The backward wave mode is extracted through post-processing the displacement field in the second material. Also, a narrow bandwidth tone burst signal consisting of 20 cycles was given, as the input, to restrict other frequency components in the sample plate. The forward and backward propagating waves have the same phase velocity, but different group velocity values. At the selected frequency the S_2_ mode in Aluminium and S_2b_ mode in Molybdenum have the same wavelength. Displacement continuity was enforced at the interface between the two materials. The wave propagation parameters at a frequency of 1.09 MHz are listed in Table 2.

**Table 2:** Parameters of the modes of interest used for the calculation of wavelength for different Lamb modes in the Material Contrast lens.

Mode	Material	Phase Velocity (m/s)	Wavelength (mm)
S_2_	Aluminium	52360	48
S_2b_	Molybdenum	52360	48
S_1_	Molybdenum	7730	7.1

**Figure 2:**
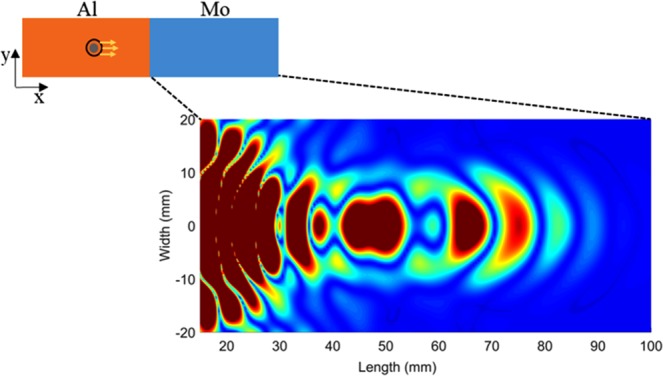
Simulation results showing focusing observed in the Molybdenum section of Al-Mo material contrast lens (see inset for schematic).

It is clear from the above table that, there is a large difference in the phase velocities of S_1_ and S_2b_ of Molybdenum, due to the highly dispersive region at the selected frequency. The ‘out-of-plane’ displacement extracted from simulation of wave propagation through the Material Contrast lens at selected frequency was filtered in the wavenumber domain at a given instant of time, to reveal the presence of backward propagating mode presence in the wave field. The focal spot can be clearly seen at a distance of 50 mm from the interface on the Molybdenum side of the lens as shown in Fig. 2.

### Experimental Validation

**Figure 3:**
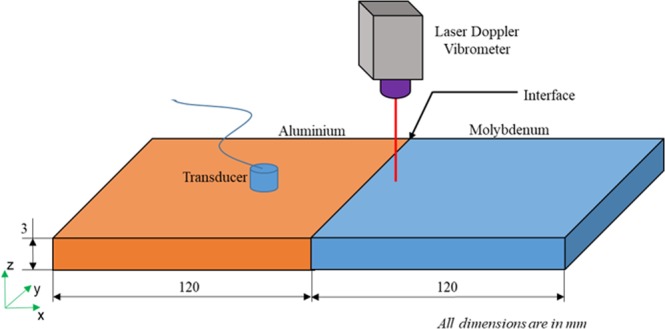
Schematic of the experimental setup. Thickness of proposed lens is 3 mm, the thickness at which S2 of aluminium intersects with S2b of Molybdenum.

The schematic of experimental configuration to demonstrate the concept of proposed material contrast lens is shown in Fig. 3. The figure depicts an Aluminium-Molybdenum (Al-Mo) material contrast lens excited using a commercially available 1 MHz PZT transducer, and it generates the possible in-plane modes in the Aluminium plate. The S2 mode was not selectively excited during the experiment (more details in Experiment setup section). At the interface between two materials, couplant gel was applied to ensure transmission of waves from Aluminium to Molybdenum. In addition, a force is applied on both ends, to remove any air gaps between the two plates.

The waves transmitted across the interface were received using a Laser Doppler Vibrometer. When the forward propagating S_2_ mode comes across the interface, a part of it is reflected, while the transmitted portion of the wave gives rise to the backward propagating S_2b_ mode. The out-of-plane displacement at each point on the surface of the lens was captured. The acquired displacement field was then processed further for filtering the modes based on wave number. In another experiment, for comparison purposes, the Molybdenum part in the material contrast lens was replaced by an Aluminium section of the same dimensions, yielding a single Aluminium-Aluminium joined plate (hereafter referred to as the ‘Al-Al’ plate).

**Figure 4:**
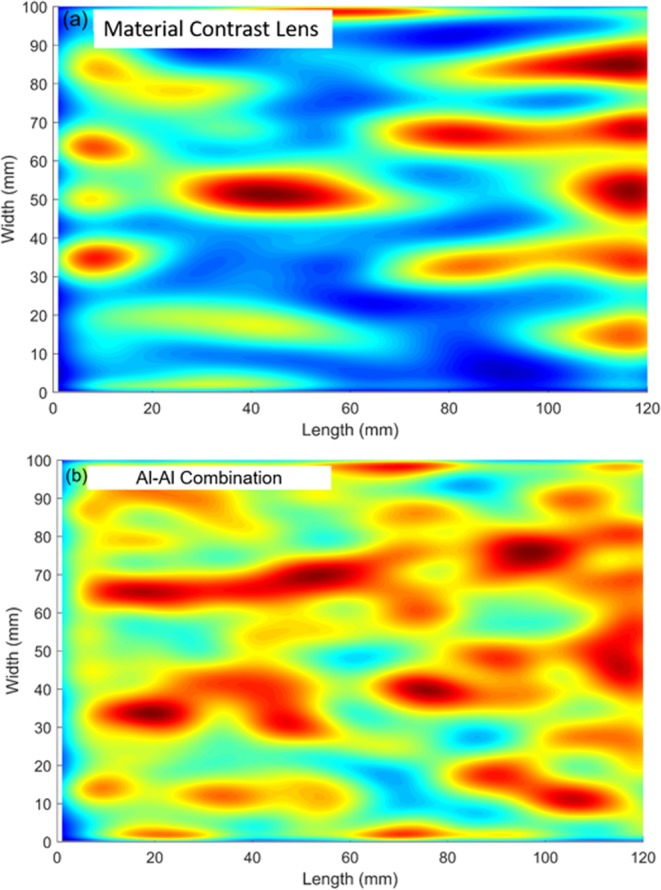
Top View. 2D scan representation obtained from experimentally monitored wave amplitude on the surface of plate across the interface. **(a)** Material contrast lens **(b)** Al-Al combination. Focusing is observed at 47 mm from the interface in material contrast lens compared to Al-Al combination.

Figure 4 represents the acquired wave field at an instant of time. The data is processed in a similar way as explained above in the section on numerical simulation, to isolate the backward mode. The comparison of results from these two cases is shown in Fig. 4(a,b). Figure 4(a), shows that the waves are focused in material contrast lens at a distance of 47 mm from the interface or at 167 mm from the excitation end, when compared to Al-Al combination, as shown in Fig. 4(b), where there is no focusing is observed.

**Figure 5:**
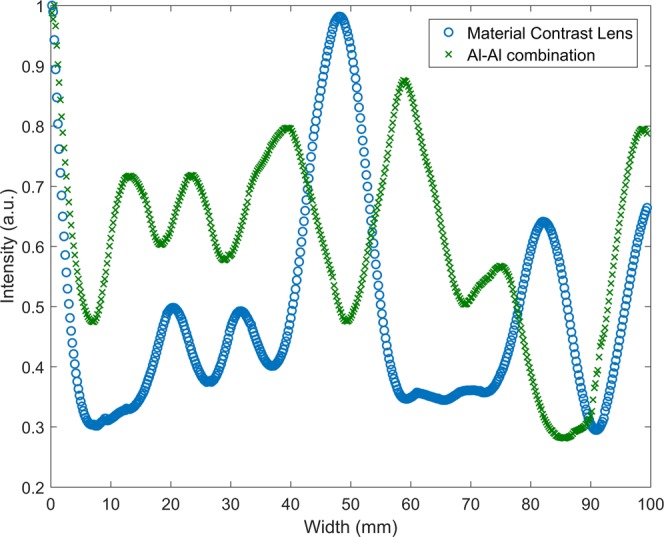
Plot showing the variation of the measured out-of-plane displacement amplitude along the width for material contrast lens compared with Al-Al combination.

Further, out -of -plane displacements were acquired along the width of the specimen across the focal spot, i.e., at a distance of x = 47 mm from the interface in the material contrast lens to confirm the focusing effect. Focusing can clearly be observed at the center of the plate. This distribution of wave energy was compared with that in case of Al-Al combination. In the latter case, the energy is more distributed as can be seen in Fig. 5.

**Figure 6:**
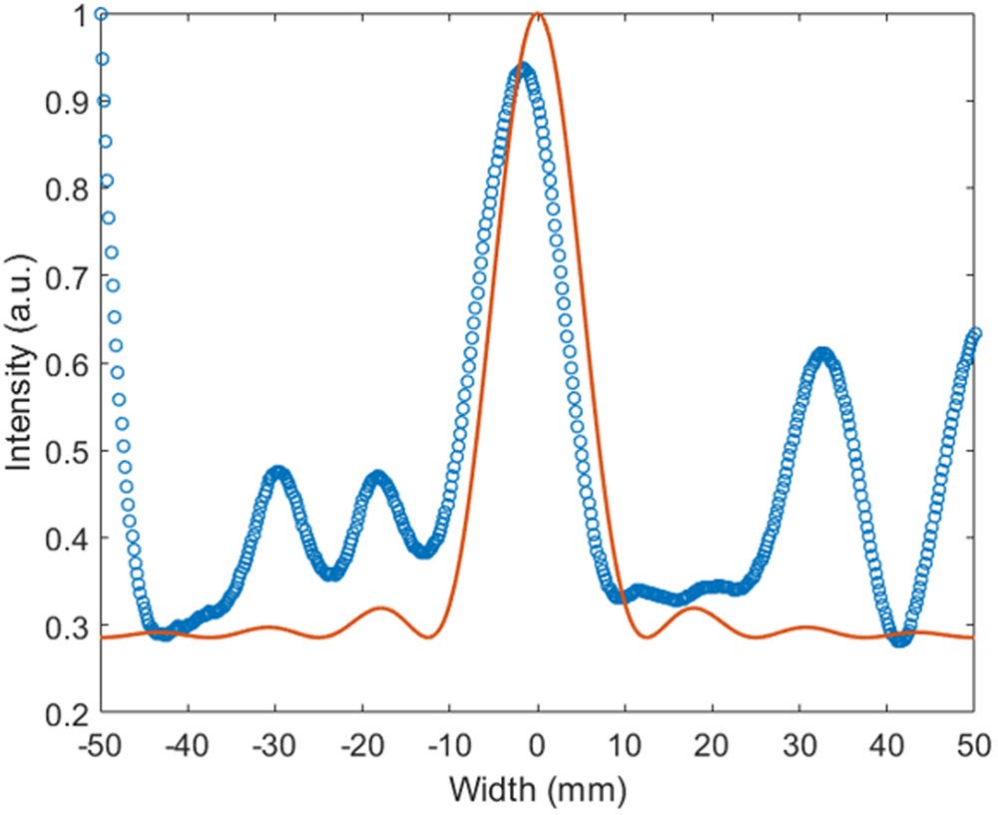
The normalized intensity distribution across the focal spot in the material contrast lens, experimental data shown in circles and solid line shows data fit according to Equation 3.

From standard diffraction theory, the intensity of each point is given by^24^,

3 $$I\propto {(\frac{\sin (ka\sin (\theta ))}{ka\sin (\theta )})}^{2}$$

where, *k* is wave number, sin(*θ*) is numerical aperture, *a* is the lateral distance from the focal point. The data acquired in the experiments is in accordance with the proportionality given by Equation 3, as can be seen in the Fig. 6. The Full Width at Half Maximum (FWHM) in the lens is 40 mm and the wavelength of the selected mode is 48 mm. The diameter of the focal spot in the material contrast lens is close to the diffraction limit of λ/2.

## Discussion

The signals acquired at every point along the scan (monitored within the Molybdenum portion) are time-gated to avoid multiple reflections within the lens. We observe from Fig. 4, that the lens achieves a region of maximum amplitude concentrated at a distance of 47 mm from the interface. The waves transmitted across the interface get converted to backward moving waves leading to focusing. Higher order symmetric modes S_1_, S_2_ are also generated along with the fundamental symmetric S_0_ mode at the selected frequency. Due to this the backward propagating mode S_2b_ is masked by the lower order modes. To observe the focusing, other modes are filtered in the wavenumber domain.

The amplitude variation along the width in the material contrast lens is symmetric about the center, as shown in Fig. 5. In case of the Al-Al configuration, the amplitude variation across the specimen is more spread out. This is expected as there is no impedance mismatch between the two sides of the interface and most of the waves get transmitted without any refraction. The significant amplitude increase is observed in the material contrast lens at the focal spot, which is higher in magnitude than that observed at same location in the Al-Al configuration. This cannot be entirely due to geometric dispersion as the incident wave propagates with a plane wave front.

The increased strength of the signal amplitude in the material contrast case can be attributed to the fact that at the interface, negative refraction is caused by the conversion of the S_2_ mode into S_2b_, which propagates backward within Molybdenum with a negative group velocity. This results in energy entrapment of the S_2b_ mode and leads to formation of a focal spot. Hence the signal amplitude at this spot is significantly higher than that of the Al-Al configuration.

An observation of the above results also shows that there are aberrations behind (before) the focal spot, which could be a point of concern. Perhaps these are artifacts of any method that induces abrupt changes in acoustic impedance across an interface, since such aberrations have also been observed with Step Change lenses^23,24^. A gradual change in the impedance at the interface may help reduce this; such studies are beyond the scope of the present report focused on a first demonstration of the concept of Material contrast lenses. However we believe that such aberrations will not hinder the ability to image features of interest beyond a certain ‘dead zone’, especially if the lens can image sub-wavelength features and achieve super-resolution. Since very little literature is available on imaging using Step Change lenses, we take support from the evidence of effective imaging using Phononic Crystals (PCs), despite a stronger field of aberrations behind the focal spot in this case, as the amplitude of these ‘aberrant’ waves attenuates rapidly after the interface^14,17^. Other recent developments^25–27^ in the context of acoustics also provide possible avenues for exploration in overcoming such challenges by extension to the ultrasonic domain.

**Figure 7:**
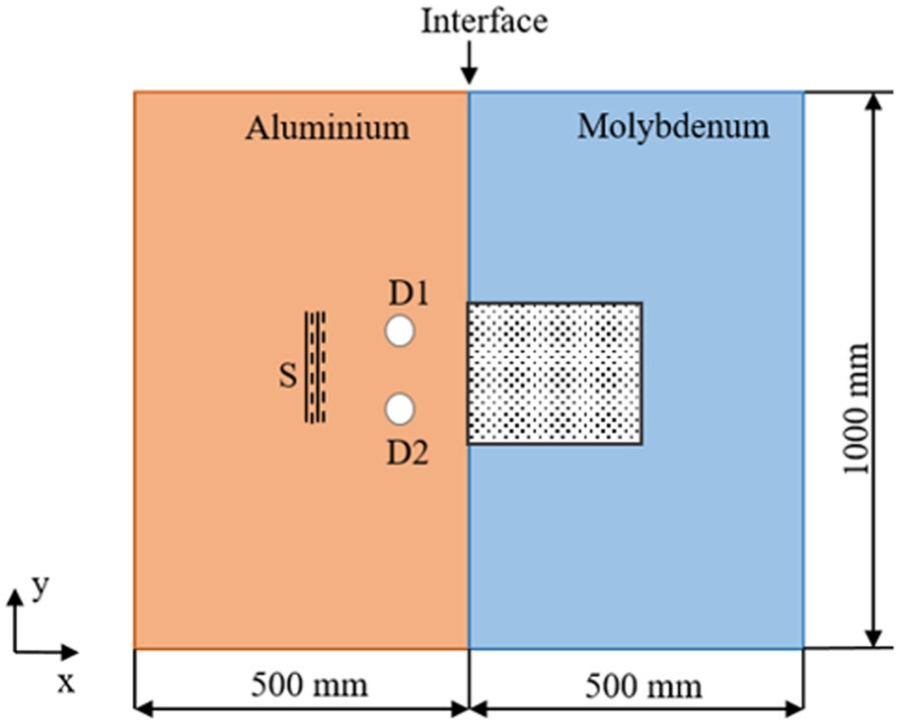
Schematic representation showing the configuration used to study the subwavelength imaging of defects using numerical simulations. S is the plane wave source, D1 & D2 are the defects, which are in subwavelength regime and placed near the interface of the material contrast lens. The dotted area shown  on the Molybdenum side is the region selected for imaging of defects.

In order to further demonstrate this, we performed additional FE simulations to try and image circular defects separated by a subwavelength distance (λ/3). Figure 7 shows the top view (schematic) of the material contrast lens, in which the defects are separated by a distance of λ/3, where λ is the operating wavelength.

**Figure 8:**
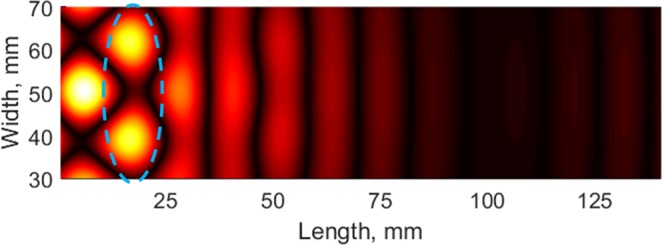
Simulation results showing the imaging of the defects in subwavelength regime on Molybdenum side of the material contrast lens.

The defects are placed at a distance of λ/2 from the interface of the lens. To avoid the reflections from the edges of the plate, the dimensions of the plate were chosen to be sufficiently large. A plane wave source, at 1.09 MHz, is positioned at a distance approximately 1.5λ from the interface. Displacements on the surface of Molybdenum are monitored to study the imaging ability of the lens in subwavelength regime. The out-of-plane displacement acquired on the top surface of the plate was spatially filtered (for the selected region, dotted area, as shown in Fig. 7) to observe the defects. The result at a particular time instant (at 35 μs) is shown in Fig. 8. In the processed image, we can observe higher amplitudes at a distance of λ/2 from the interface (marked with an ellipse in Fig. 8) on Molybdenum side. A distance of λ/3 separates the amplitude-peaks, thus demonstrating super-resolution imaging by the proposed material contrast lens.

In conclusion, thickness change in plates has already been shown to improve the resolution in imaging using ultrasound^23,24^. Our results demonstrate for the first time, lensing in the ultrasonic domain by a combination of different materials. That the novel concept of material contrast lens that can be used to focus ultrasonic waves has been demonstrated numerically and validated through experiment. The focusing is achieved by the mode conversion of S_2_ to S_2b_, while it travels from Aluminium into Molybdenum. Super-resolution imaging using the proposed lens is demonstrated using numerical simulations. The proposed lens is suitable in all applications where higher resolution ultrasonic imaging is of much interest, such as biomedical diagnostics and non-destructive evaluation of engineering components. Material contrast lenses could for example be used in conjunction with conventional ultrasonic probes to achieve super-resolution. Also, similar to the application of Phononic Crystals, the focusing by material contrast lens could also find application in energy harvesting devices^39^. Further, such a material contrast arrangement can be readily miniaturized, implemented in lensing devices, and also eventually used to create lens-stacks, which is the focus of ongoing work in our group.

## Methods

### Simulation

Numerical simulations were carried out using commercially a available finite element package^40^. The simulation was carried out to study the behavior of the wave displacement field in  the three-dimensional domain. The material properties given in Table 1 were used in the simulation. The lens was modeled using eight-noded brick elements (C3D8R – 3D Stress). The increment time step was selected based on criterion given in^41^. In-plane excitation consisting of a Hanning windowed tone burst signal of 20 cycles, defined by $0.5[1-\,\cos (\frac{2\pi ft}{n})]\cos (2\pi ft)\,\,$was given as input was given along the width of the plate, where *f* is the excitation frequency, *t* is the pulse duration and *n* is number of cycles. The out-of-plane displacement field of each node was extracted from the required region. This data was post processed using 2D Fast Fourier Transform (FFT). A 2D Gaussian filter was used and the data field was filtered based on wavenumber of 0.028 mm^−1^. The dimensions of the plate were chosen in such a way to avoid the reflections from the lateral sides.

### Experiment Setup

Experiments were performed on the material contrast lens using pitch catch ultrasound technique. The ultrasonic waves were generated using a commercially available contact transducer (Panametrics–GE Measurement and Control, Billerica, Massachusetts, USA) at the desired frequency of 1.09 MHz. A 20 cycle, Hanning windowed tone-burst input is applied at the transducer by means of a RITEC 4000 Pulser- Receiver (RITEC Inc., Warwick, Rhode Island, USA).

### Measurement of wave displacement filed in experiment

The displacements were measured using a Fiber-Optic Laser Doppler Vibrometer (OFV 551, Polytec GmbH, Germany) controlled by a Laser Controller (OFV 5000, Polytec). A retroreflective tape was pasted uniformly over the monitoring surface in order to obtain better output, which was digitized and time averaged at 512 signals using a digital storage oscilloscope (DSO-X-4104A, Keysight Technologies, USA). The scans were performed using a 3-axis automated scanning system, operated by a motion controller (MID 7604, National Instruments) and data was acquired using LabVIEW (2016 version, National Instruments). The scanning speed was maintained at 0.5 mm/s in order to ensure proper sampling and acquisition.

## Data Availability

The datasets generated during and/or analysed during the current study are available from the corresponding author on reasonable request.
